# Clinical Pharmaceutical Reasoning in Hospital Pharmacy Practice using the DRIP framework: a New Approach for a Perfectionist Profession

**DOI:** 10.12688/mep.20468.1

**Published:** 2024-07-15

**Authors:** Heleen van der Sijs, Midas B. Mulder

**Affiliations:** 1 Hospital Pharmacy, Erasmus MC, University Medical Center, Rotterdam, PO Box 2040, 3000 CA, The Netherlands; 2 Clinical Pharmacy, Haaglanden Medical Center, The Hague, Lijnbaan 32, 2512 VA, The Netherlands

**Keywords:** clinical reasoning, clinical pharmacy, medication safety, pharmacy education

## Abstract

Clinical pharmacists are responsible for safe medication use in hospitals. Most clinical pharmacists are perfectionists. However, in their decision-making process, they have to embrace uncertainty, while interpreting available data, and integrating knowledge and clinical experience. In clinical practice, how to teach and master clinical pharmaceutical reasoning is unclear.

We developed the DRIP framework including different aspects on drug, indication and patient and a stepwise approach to support clinical pharmaceutical reasoning by students, residents in hospital pharmacy and clinical pharmacists.

The DRIP framework was first introduced during the daily report with residents and faculty of the clinical pharmacy. The framework was implemented in daily clinical practice to handle drug safety alerts, and to optimise drug therapy during ward rounds, multidisciplinary consultations, and in entrustmentbased discussions with residents.

Pharmacists using the DRIP framework felt more confident that relevant aspects of a complex pharmaceutical problem had been considered, they learned to anticipate on the issues behind an apparently simple pharmaceutical question, and to explain their reasoning. Several CANMEDS roles can be simultaneously trained by using our approach.

We are developing a course for residents in clinical pharmacy and pharmacology to teach the competency of clinical pharmaceutical reasoning using the DRIP framework.

## Practice points

- Clinical pharmacists are perfectionists that have to embrace uncertainty
- How to teach clinical pharmaceutical reasoning is unclear
- The DRIP framework shows aspects of drug, indication and patient to consider
- The accessory stepwise approach supports the clinical pharmaceutical reasoning process

## Educational challenge

Clinical pharmacists are responsible for the safe use of medications in hospitals. Therefore, they are trained to be perfectionists to avoid risks associated with medication. Healthcare has become more complex over the years because of multimorbidity, polypharmacy, and shorter hospital stays of severely ill patients. Thus, clinical pharmacists must deal with guidelines that conflict with or are not applicable to patients at hand. Moreover, they have to accept and embrace complexity and uncertainty and weigh the risks and benefits using relevant context parameters in their decision-making process. Clinical pharmaceutical reasoning is defined as a context-dependent cognitive process whereby pharmacists apply and integrate knowledge and clinical experience to interpret the available clinical data ( Mertens *et al.*, 2022).

Clinical pharmaceutical reasoning is often necessary when questions seem simple and straightforward, such as questions about the right dose, route of administration, or potential interaction between two or more drugs. In general, context parameters must be considered to formulate an adequate answer. For example, the rather simple question ‘Can this drug be administered through a feeding tube?’ generally requires more information on the patient, the reason for prescribing (indication or diagnosis), and the drug (regimen). How old is the patient, are there swallowing problems, and which administration form and dosing schedule are prescribed? The answer ‘no’ does not help the nurse or physician and will result in a follow-up question.

Thus, clinical pharmaceutical reasoning is of vital importance in clinical pharmacists’ daily practice, and this complex skill should be developed and taught. It is also a trust criterion for Dutch residents in clinical pharmacy to grant them entrustable professional activities (EPAs) ( Ten Cate & Hoff, 2017). Residents should not only demonstrate that an adequate answer has been given in a certain case but also how context-dependent risks and benefits are weighed with different context parameters in a so-called entrustment-based discussion. Thus, supervisors should be able to understand and express their own decision-making processes beyond their gut feelings. However, pharmacists lack training in clinical reasoning, as opposed to physicians, who are intensively trained in clinical diagnostic reasoning at medical schools ( Woodruff, 2019).

We recognized that pharmacy students and clinical pharmacy residents relied too much on published case reports, did not consider all relevant context parameters in their recommendations, and could not explain their reasoning. Moreover, supervisors do have difficulties in explaining their clinical decision-making process to students and residents.

## What was the solution?

**Figure 1.**
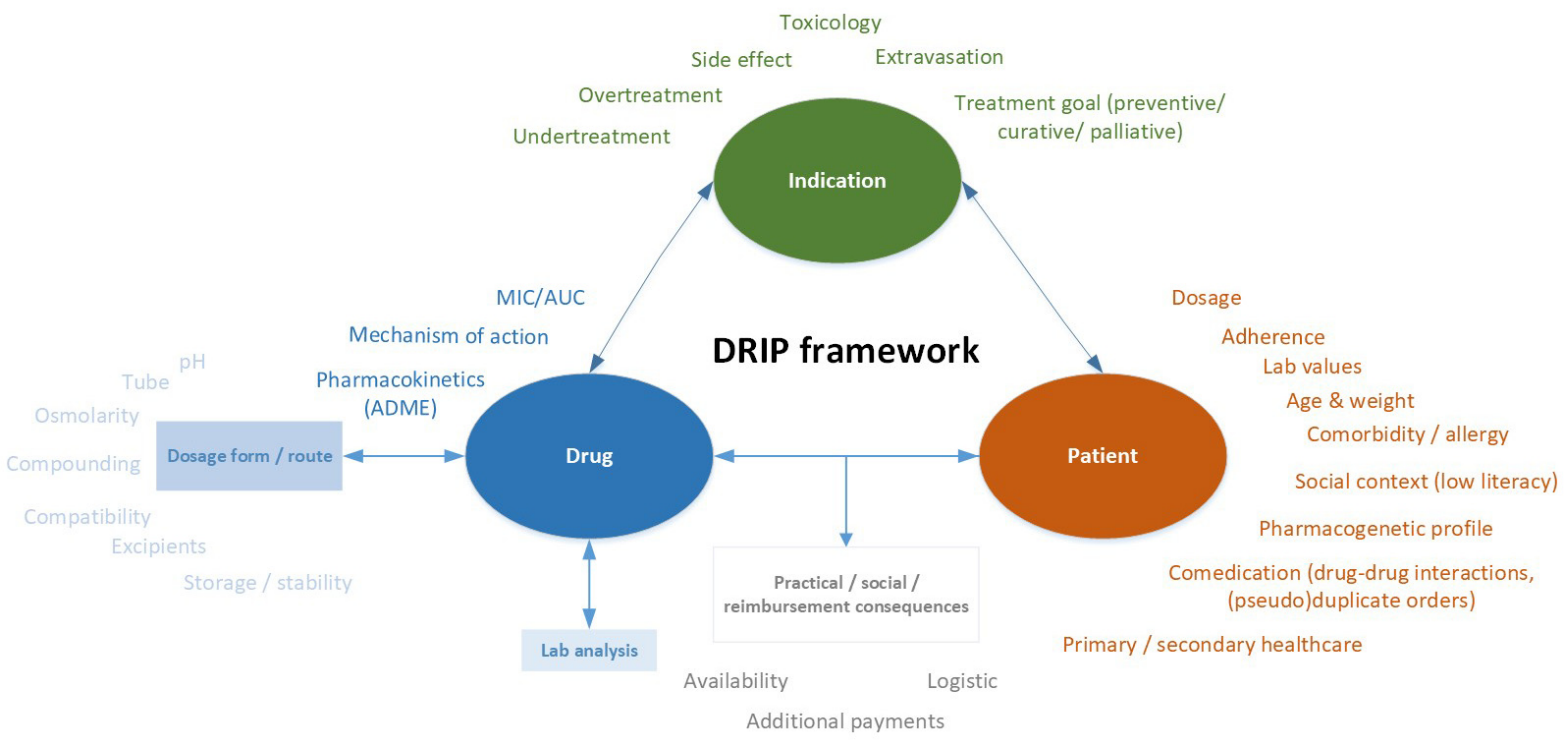
DRIP framework for clinical pharmaceutical reasoning.

In 2021, we developed a framework for clinical pharmaceutical reasoning. This DRIP framework includes many relevant aspects of **dr** ug, **i** ndication, and **p** atient that may influence the responses of clinical pharmacists to medication-related questions and tasks (See Figure 1). The framework is not comprehensive in that it can be used as a checklist/roadmap. It is merely a toolbox that should be used with adaptive capacity and professional values (e.g., accountability, moral integrity, trustfulness) ( Woodruff, 2019). With increasing complexity, people are inclined to use a reductionist standardized approach that is not applicable to complex systems. The framework may provide insight into unknown aspects that should be taken into consideration. Acknowledging and embracing this uncertainty may result in better clinical reasoning ( Stolper *et al.*, 2021). The DRIP framework focuses on content, but this is not sufficient to support the clinical reasoning process that requires the integration of relevant data ( Mertens *et al.*, 2022). 

To provide guidance in the clinical reasoning process, we developed a stepwise approach next to the DRIP framework, including the following steps: (a) summarize the medication problem/question, (b) map all relevant patient and drug characteristics and consult trustworthy information sources; (c) interpret lab values and patient parameters taking into account context, history, and comedication; (d) weigh the benefits and risks of possible solutions, including implications of stopping drugs on drug-drug interactions and therapeutic drug monitoring, (e) test for feasibility on practical, financial, social, and sustainability levels; (f) discuss the options with the healthcare professional, conclude and record in the patient file, and (g) follow-up and learning.

## How was the solution implemented?

Our DRIP framework for clinical pharmaceutical reasoning has been introduced and used in our daily clinical pharmacy report. In case-based discussions during the daily report, questions were asked using the framework, for example, ‘What if the patient was pregnant or obese?’, or ‘What would you recommend if the intoxicated patient was seen within one hour of ingestion, or ingested sustained release tablets?’. Residents were invited to answer first and explain their clinical pharmaceutical reasoning process, but the questions also stimulated this process among faculty and pharmacy students. The framework was evaluated and did not require any adjustment. The printed DRIP framework was visible in the room for daily reporting and digitally linked to daily report meetings. 

In addition to their use in daily reports, residents and consultant clinical pharmacists were encouraged to use this framework during their clinical activities and on-call shifts to serve clinics with well-considered recommendations. The framework was applied during the daily rounds on the wards, in multidisciplinary consultations, in one-to-one supervision of residents in handling drug safety alerts, in entrustment-based discussions with residents, and in the supervision of pharmacy students. 

## What lessons were learned that are relevant to a wider global audience?

By introducing the DRIP framework, our students, residents, and consultant clinical pharmacists were taught to provide a more holistic overview of the complexity of clinical pharmaceutical problems. We recognize that the DRIP framework may unveil unknown aspects and, consequently, whether the case is simple or complex ( Stolper *et al.*, 2021). Consultant clinical pharmacists and senior residents said that they used the DRIP framework only for complex cases, and novices perceived it more often as helpful. The stepwise approach was mainly used by students and less experienced pharmacists. Although all the steps must be completed, the decision-making process is often not straightforward. Particularly in complex cases, the steps occur crisscrossing and iteratively arrive at the final recommendation ( Woodruff, 2019). However, using the stepwise approach ensures that all relevant steps are completed, including practical consequences to anticipate further questions and learning by performing follow-ups.

The most important lessons learned from the implementation of the DRIP framework for clinical pharmaceutical reasoning are as follows.

- Pharmacists felt more confident that the relevant aspects of a difficult and complex pharmaceutical problem were considered.
- Pharmacists could better explain their reasoning process;
- Pharmacists learned to anticipate the issues behind the apparently simple pharmaceutical questions of healthcare workers or patients.

Furthermore, the DRIP framework appeared to be helpful in entrustment-based discussions by varying one or several aspects, and in the simultaneous training of the CanMEDS roles of communicator, collaborator, professional, and scholar.

## What are the next steps?

The first step in developing a tool for clinical pharmaceutical reasoning in clinical pharmacy resulted in a well-received DRIP framework and an additional stepwise approach. Currently, we are developing a course for the national education program for residents of clinical pharmacies and their (deputy) trainers using the DRIP framework. As awareness of the lack of training of Dutch pharmacists in clinical pharmaceutical reasoning is growing, we have tried to build collaborations with other universities, teaching hospitals, and pharmacy schools in the Netherlands.

As prescribers may also benefit from the DRIP framework, and both physicians and pharmacists may learn from each other, we would like to expand to an interprofessional clinical pharmaceutical reasoning course for fellows of the Dutch Society for Clinical Pharmacology and Biopharmacy.

## Ethics and consent

Ethical approval and consent were not required.

## Disclaimer

The views expressed in this article are those of the authors. Publication in MedEdPublish does not imply endorsement by MedEdPublish.

## Data Availability

No data associated with this article.
