# Supplementary material for: Clinical Pharmaceutical Reasoning in Hospital Pharmacy Practice using the DRIP framework: a New Approach for a Perfectionist Profession
Source: MedEdPublish (2016). 2025 Oct 24;14:56. Originally published 2024 Jul 15. [Version 2] doi: 10.12688/mep.20468.2 (PMC12596546; doi:10.12688/mep.20468.2)
Supplement: Peer review history

# Reviewer report: Reviewer response for version 2

**DOI:** 10.21956/mep.22864.r44155

**Published:** 2025-11-10

**Authors:** Tayseer Mansour

**Affiliations:** 1 Family & Community Medicine and Medical Education, Taibah University, Medina, Al Madinah Province, Saudi Arabia; 2 Medical Education, Suez Canal University Faculty of Medicine, Ismailia, Ismailia Governorate, Egypt

In this manuscript, the authors argue that clinical pharmacists, despite being perfectionists who must ensure medication safety, receive insufficient training in clinical reasoning compared to physicians. This deficiency becomes problematic when pharmacists must make complex, context-dependent decisions that require embracing uncertainty and weighing multiple factors. To address this challenge, the authors developed the DRIP framework, which systematically considers aspects related to Drug, Indication, and Patient. The framework is accompanied by a seven-step reasoning process that guides pharmacists from problem identification through follow-up and learning. The authors describe the implementation of the DRIP framework at Erasmus MC University Medical Center, where it has been integrated into daily clinical pharmacy reports, ward rounds, multidisciplinary consultations, and resident supervision. Based on subjective feedback, pharmacists report increased confidence in addressing complex pharmaceutical problems, improved ability to explain their reasoning, and better anticipation of issues underlying apparently simple questions. The authors are developing a formal course for clinical pharmacy residents and plan to expand the approach to interprofessional education.

 The DRIP framework is clearly articulated, well-visualized, and addresses a genuine educational need. However, there is one area that requires enhancement:

 - Referencing Differing Views and Opinions: While the authors compare the DRIP framework to existing tools (SOAP and PPCP), the discussion of alternative approaches or differing perspectives on clinical pharmaceutical reasoning is limited. The manuscript would be strengthened by acknowledging other frameworks or approaches to teaching clinical reasoning in pharmacy education. For example, the authors could discuss whether other institutions have developed similar tools, how clinical reasoning is taught in other pharmacy programs, or whether there are alternative perspectives on the best approach to teaching this competency. Additionally, the authors could address potential criticisms or limitations of their approach, such as whether the framework might be too complex for novices or whether it could inadvertently promote checklist-based thinking rather than adaptive reasoning. Including a brief discussion of these alternative views would provide readers with a more balanced perspective and strengthen the scholarly rigor of the open letter. This could be addressed by adding one or two paragraphs discussing alternative approaches in the literature and acknowledging potential limitations or alternative perspectives on the DRIP framework.

Is the rationale for the Open Letter provided in sufficient detail?

Yes

Does the article adequately reference differing views and opinions?

Yes

Are all factual statements correct, and are statements and arguments made adequately supported by citations?

Partly

Is the Open Letter written in accessible language?

Yes

Where applicable, are recommendations and next steps explained clearly for others to follow?

Yes

Reviewer Expertise:

Health Professions Education, Interprofessional Education, Students' Assessment, Curriculum Design

I confirm that I have read this submission and believe that I have an appropriate level of expertise to confirm that it is of an acceptable scientific standard.

# Reviewer report: Reviewer response for version 2

**DOI:** 10.21956/mep.22864.r44070

**Published:** 2025-11-08

**Authors:** Devi Prasad Mohapatra

**Affiliations:** 1 Jawaharlal Institute of Postgraduate Medical Education and Research, Pondicherry, India

The authors have presented an open letter on the use of a newly developed DRIP framework to enhance clinical pharmaceutical reasoning in a hospital setup. The manuscript reads well and will be useful for future researchers to work in this dimension.

Is the rationale for the Open Letter provided in sufficient detail?

Yes

Does the article adequately reference differing views and opinions?

Partly

Are all factual statements correct, and are statements and arguments made adequately supported by citations?

Yes

Is the Open Letter written in accessible language?

Yes

Where applicable, are recommendations and next steps explained clearly for others to follow?

Yes

Reviewer Expertise:

Clinical reasoning, critical thinking, health professions pedagogy, competency based medical education

I confirm that I have read this submission and believe that I have an appropriate level of expertise to confirm that it is of an acceptable scientific standard.

# Reviewer report: Reviewer response for version 1

**DOI:** 10.21956/mep.21906.r42315

**Published:** 2025-08-13

**Authors:** Devi Prasad Mohapatra

**Affiliations:** 1 Jawaharlal Institute of Postgraduate Medical Education and Research, Pondicherry, India

Thank you for the opportunity to review your open letter describing the **DRIP (Drug, Indication, Patient) **framework aimed at supporting clinical pharmaceutical reasoning among pharmacy residents and their trainers. The manuscript is well written. The model suggests a clear and structured approach that is easy to remember and potentially valuable for guiding therapeutic decision-making in daily practice. It highlights essential elements of rational pharmacotherapy and encourages contextual thinking.

 However, the manuscript would benefit from the inclusion of objective data demonstrating the framework’s effectiveness in practice. Statements like ‘Pharmacists felt more confident…’ or ‘Pharmacists could better explain…’ etc. are very subjective. If possible, some objective data especially before and after implementing this framework would add value to this manuscript.

 Additionally, a comparison with existing clinical reasoning tools eg; SOAP format (or similar), or pharmaceutical care process models, or reflective case-based learning method would provide a clearer basis for the reader to understand the specific features and possible benefits of the DRIP framework.

Is the rationale for the Open Letter provided in sufficient detail?

Yes

Does the article adequately reference differing views and opinions?

Partly

Are all factual statements correct, and are statements and arguments made adequately supported by citations?

Yes

Is the Open Letter written in accessible language?

Yes

Where applicable, are recommendations and next steps explained clearly for others to follow?

Yes

Reviewer Expertise:

Clinical reasoning, critical thinking, health professions pedagogy, competency based medical education

I confirm that I have read this submission and believe that I have an appropriate level of expertise to confirm that it is of an acceptable scientific standard, however I have significant reservations, as outlined above.

# Response

**Published:** 2025-10-22

**Authors:** Heleen van der Sijs

**Affiliations:** Hospital Pharmacy, Erasmus Medical Center, Rotterdam, South Holland, The Netherlands

1. Thank you for your suggestion to include data on the framework's effectiveness in practice. Unfortunately, we did not evaluate the DRIP-framework with a structured questionnaire. However, we are currently working on a project to evaluate Dutch pharmacists's uncertainty tolerance in relation to the use of the DRIP-framework. After the first pilot course on clinical pharmaceutical reasoning, 9 out of 16 participants were positive about the framework and would implement it in their current clinical practice. 2. Thank you for the suggestion to compare the DRIP-framework with other clinical reasoning tools. We have added a comparison with SOAP and PPCP in the second version of the article.

# Reviewer report: Reviewer response for version 1

**DOI:** 10.21956/mep.21906.r39008

**Published:** 2024-10-17

**Authors:** Sarah Alghanem

**Affiliations:** 1 Kuwait University, Kuwait City, Al Asimah Governate, Kuwait

I would like to thank the authors for the paper , the idea about DRIF framework is great. however, I have few comments to improve clarity.

 1. How was the framework developed? It would be helpful to explain the process. Authors are refereeing to to paper "Mertens JF, Koster ES, Deneer VHM, et al.: Clinical reasoning by pharmacists:

 a scoping review. Curr Pharm Teach Learn. 2022; 14(10): 1326–1336.", it is a review article and not clear whether the framework was developed from that review or from a different publication?

 2. It was not clear how authors identified the lessons learned from using the framework? Was there any survey or focus group interviews?

 3. What assessment tools used to assess students reasoning? Was there a pre- post evaluation to assess the impact for the framework?

Is the rationale for the Open Letter provided in sufficient detail?

Partly

Does the article adequately reference differing views and opinions?

No

Are all factual statements correct, and are statements and arguments made adequately supported by citations?

Partly

Is the Open Letter written in accessible language?

Yes

Where applicable, are recommendations and next steps explained clearly for others to follow?

Partly

Reviewer Expertise:

Require more clarification in the aspects mentioned in the report.

I confirm that I have read this submission and believe that I have an appropriate level of expertise to confirm that it is of an acceptable scientific standard.

# Response

**Published:** 2025-10-22

**Authors:** Heleen van der Sijs

**Affiliations:** Hospital Pharmacy, Erasmus Medical Center, Rotterdam, South Holland, The Netherlands

The framework was developed from clinical expertise of the authors and was already in use before the scoping reviews of Mertens et al. had been published online. The first draft of the framework was adjusted by the authors while attending daily clinical pharmacy report sessions. Thereafter, the current version was introduced and used in these sessions.  We evaluated the (use of the) DRIP-framework within our hospital pharmacy several times by discussing it. In addition, we introduced the framework in a national workshop and asked for comments, which did not result in adjustments. Furthermore, the DRIP-framework was used in a national course for residents in hospital pharmacy in November 2024. Participants verbalized the framework was helpful. Questionnaires or focus groups were not performed. Assessment of diagnostic reasoning can be performed by comparing proposed to real diagnosis retrospectively. However, a valid assessment tool for clinical pharmaceutical reasoning is lacking, as several answers may be correct. Use of the script concordance test has serious drawbacks and using this test for a pre-post evaluations was therefore deemed inappropriate. Therefore, assessment of clinical pharmaceutical reasoning preferably takes place by experienced clinicians in (entrustment-based) discussions.
